# Echovirus 13 Aseptic Meningitis, Brazil

**DOI:** 10.3201/eid1208.051317

**Published:** 2006-08

**Authors:** Claudete I. Kmetzsch, Estela M.R. Balkie, Anita Monteiro, Eliane V. Costa, Gina P.L. dos Santos, Edson E. da Silva

**Affiliations:** * Instituto Oswaldo Cruz, Rio de Janeiro, Brazil;; † Secretaria Municipal de Saúde do Rio Grande do Sul, Rio Grande, Brazil;; ‡ Instituto de Pesquisas Biológicas, Rio Grande, Brazil

**Keywords:** Echovirus 13, Aseptic Meningitis, letter

To the Editor: Human enteroviruses (polioviruses, coxsackievirus A, coxsackievirus B, echoviruses, enterovirus 71, and newer recognized serotypes) belong to the Picornaviridae family, Enterovirus genus (*1*). They are common viral agents associated with a diversity of clinical manifestations, including respiratory illness; nonspecific rashes; hand, foot, and mouth disease; myocarditis; acute hemorrhagic conjunctivitis; and central nervous system (CNS) syndromes (*2*). Acute viral infections of the CNS are the source of a group of globally distributed diseases, which affect the population in a sporadic, endemic, or epidemic way. These infections cause a number of illnesses, particularly in children, and may result in serious sequelae; in severe cases, they can be fatal (*3*). Meningitis, encephalitis, acute flaccid paralysis (poliomyelitis), mononeuritis, polyneuritis, and Reye syndrome constitute most of the illnesses (*4*). Nonpolio enteroviruses are responsible for >80% of viral meningitis cases in which the etiologic agent is identified (*2*). Several of the 28 currently recognized serotypes of echovirus are found in association with these infections (*3*).

We describe an outbreak of aseptic meningitis that occurred in southern Brazil in 2003 with echovirus 13 (E13) virus as the etiologic agent. This is the first meningitis outbreak due to E13 reported in the country.

From March to April 2003, 17 children and young adults from Horizontina City (population 16,800), Rio Grande do Sul State, southern Brazil, with symptoms of meningitis, sought medical attention at the local hospital. Seven of these case-patients were linked to each other either by school or domiciliary contact. Lumbar puncture showed clear cerebrospinal fluid (CSF), which suggests a viral cause. The following symptoms were associated with patients: fever (92%), headache (84%), vomiting (79%), diarrhea, stiff neck, and fatigue (7.69% each). Patients' ages ranged from 1 to 19 years of age, with the age peak incidence in children 5–9 years of age (46%). Fifty-eight percent of patients were male. All patients recovered, and no sequelae or deaths were identified. The pattern of meningitis associated with E13 in this outbreak was clinically similar to those observed in aseptic meningitis due to other enteroviruses in previous outbreaks.

For diagnostic purposes, 12 CSF and 8 fecal specimens were collected from the 17 patients with clinically suspected viral meningitis. For viral diagnosis, RD and HEp2 cells were injected with 0.2 mL of each clinical specimen (clarified fecal specimens and CSF) and examined daily for at least 7 days postinoculation. Enterovirus characteristic cytopathic effect was observed in 6 (50%) of 12 CSF samples and in 5 (62.5%) of 8 fecal samples. All isolates were typed as echovirus 13 by a reverse transcription–PCR and nucleotide sequencing of a portion of the VP1 gene (*5*).

Before 2000, echovirus 13 was considered a rare serotype of enterovirus (*6*) and had never been reported in association with outbreaks (*7*). In the United States, before 2001, this enterovirus accounted for only 65 of the 45,000 reported enteroviral isolates (*6*). However, the incidence of E13 is increasing; several meningitis outbreaks have been recently reported in England, Germany, Belgium, Spain, France, Israel, and Japan (*8*).

In spite of the temporal clustering and close contact of 7 patients, the causes of the outbreak were not completely defined and remain speculative. The sudden emergence of E13 as a prominent enterovirus associated with viral meningitis in many countries, including Brazil, demonstrates the potential of enteroviruses to circulate widely and to unpredictably cause diseases, which underscores the continued need for enterovirus surveillance.

Although this specific outbreak was restricted both geographically and in terms of magnitude (only 17 cases), E13 seemed to be widely distributed in Brazil and has been detected in fecal specimens obtained from patients with acute flaccid paralysis since 1998 (C. Blal, unpub. data). Epidemiologic surveillance plays a crucial role in understanding the changing patterns of enterovirus infection and disease associations. Such knowledge may help in the control of diseases (*9**,**10*). Although identifying the enterovirus serotype does not contribute substantially to patient management, establishing the dominant virus each year or in each outbreak is essential for epidemiologic purposes.
